# Risk Factors for Obesity: Further Evidence for Stronger Effects on Overweight Children and Adolescents Compared to Normal-Weight Subjects

**DOI:** 10.1371/journal.pone.0015739

**Published:** 2011-01-20

**Authors:** Andreas Beyerlein, André M. Toschke, Angelika Schaffrath Rosario, Rüdiger von Kries

**Affiliations:** 1 Institute of Social Paediatrics and Adolescent Medicine, Ludwig-Maximilians University of Munich, Munich, Germany; 2 Department of Medical Informatics, Biometry and Epidemiology and Munich Centre of Health Sciences, Ludwig-Maximilians University of Munich, Munich, Germany; 3 Robert Koch-Institute, Berlin, Germany; Brigham and Women's Hospital, Harvard Medical School, United States of America

## Abstract

**Background:**

We recently showed that in preschoolers risk factors for overweight show stronger associations with BMI in children with high BMI values. However, it is unclear whether these findings might also pertain to adolescents.

**Methods:**

We extracted data on 3–10 year-old (n = 7,237) and 11–17 year-old (n = 5,986) children from a representative cross-sectional German health survey (KiGGS) conducted between 2003 and 2006 and calculated quantile regression models for each age group. We used z-scores of children's body mass index (BMI) as outcome variable and maternal BMI, maternal smoking in pregnancy, low parental socioeconomic status, exclusive formula-feeding and high TV viewing time as explanatory variables.

**Results:**

In both age groups, the estimated effects of all risk factors except formula-feeding on BMI z-score were greatest for children with the highest BMI z-score. The median BMI z-score of 11–17 year-old children with high TV viewing time, for example, was 0.11 [95% CI: 0.03, 0.19] units higher than the median BMI z-score of teenage children with low TV viewing time. This risk factor was associated with an average difference of 0.18 [0.06, 0.30] units at the 90^th^ percentile of BMI z-score and of 0.20 [0.07, 0.33] units at the 97^th^ percentile.

**Conclusions:**

We confirmed that risk factors for childhood overweight are associated with greater shifts in the upper parts of the children's BMI distribution than in the middle and lower parts. These findings pertain also to teenagers and might possibly help to explain the secular shift in the upper BMI percentiles in children and adolescents.

## Introduction

All over the world, increasing prevalences of childhood overweight have been reported [1], [2], [3] which appear to be based mainly on a shift of the upper parts of the body mass index (BMI) distribution rather than a shift of the BMI in the whole population [4], [5].

In a previous study using data from the Bavarian school entry examinations [6], we observed that risk factors for overweight are associated with stronger effects on higher BMI percentiles than on average BMI values, suggesting that incremental exposure to those risk factors would primarily result in more extreme values of BMI or body weight. We hypothesized that these findings might help to explain the observed temporal trend in overweight and obesity: If a risk factor shows stronger effects on higher BMI values and the exposure frequency of this risk factor has increased over time, an increase of the upper BMI percentiles within a population could be explained.

However, our previous analyses [6] were based on children at pre-school age (5–6 years), and it therefore remains unclear whether these findings might also pertain to older children and adolescents for whom a similar shift of the BMI distribution affecting predominantly the upper percentiles has been observed [4], [7]. We analyzed a large German population-based dataset on children and adolescents in order to answer this question and to assess potential age-specific effects.

## Methods

The data were collected from May 2003 to May 2006 in the baseline wave of the German Health Interview and Examination Survey for Children and Adolescents (KiGGS), a representative cross-sectional nation-wide survey on children and adolescents selected within 167 communities (primary sample points). In a second step, addresses of families were drawn randomly from local registries to invite the children to participate in the survey. The response rate was 66.6% [8]. Overall, n = 17,641 children aged 0 to 17 years were enrolled. About 2/3 of the non-participants filled in a short questionnaire with a few basic questions, so that some information was available for almost 89% of the contacted population. Questions on self-reported height and weight were part of the short questionnaire. The study was approved by the Institutional Review Board of the Virchow-Klinikum of the Humboldt-University Berlin. A detailed description of the survey has been published elsewhere [8], [9].

Information on covariates and life style factors was obtained from a self-administered questionnaire from parents and also from the children themselves (in children aged 11 years and older). For non-German families, questionnaires in their native languages were provided. Maternal smoking in pregnancy was documented in three categories (never, occasionally or regularly) and dichotomised to never or any. Mothers were asked about their present height and weight, which were used to calculate their BMI at interview. Socioeconomic status (SES) was classified based on the parents' professional status, income and educational achievements and assigned to low, middle or high according to the parent with the higher status [10]. Exclusive formula-feeding (yes/no) was defined as no breastfeeding of the index child at any time as reported by the mothers. The child's TV viewing time per day was recorded in the following categories (ordinal value in brackets): none (1), 0.5 hours (2), 1–2 hours (3), 3–4 hours (4), >4 hours (5). In the 3–13 year-old children, TV viewing time was recorded separately for working days and weekends, while the 14–17 year-olds were only asked about their “mean” TV viewing time without differentiation between working days and weekends. We summed the values of working days and weekend TV viewing time up and defined high TV viewing time as the respective upper age-specific quartile of the observed TV viewing time (summary) score in children aged 3–6, 7–10, 11–13. In 14–17 year-olds, high TV viewing time was defined as lying within or above the upper quartile of “mean” TV viewing time. Although formally defined by age-specific quartiles, the prevalence for high TV viewing time was about 35% in both the younger and older group of children. Child's use of computer / internet was assessed in the same way, and we defined high frequency of computer use analogously. However, the group of 11–17-year-old children was additionally asked about their frequency of game pad use. To avoid bias by different assessment of computer / game pad use in different age groups, we did not consider this variable in our main analyses.

Children's height was measured, without wearing shoes, by trained staff with an accuracy of 0.1 cm, using a portable Harpenden stadiometer (Holtain Ltd., Crymych, UK). Body weight was measured with an accuracy of 0.1 kg, wearing underwear, with a calibrated electronic scale (SECA, Birmingham, UK). These measures were used to calculate children's BMI. To adjust children's BMI for sex and age, we transformed the observed BMI values to sex- and age-specific z-scores established by the World Health Organisation (WHO) (http://www.who.int/growthref/en) [11].

We excluded 2,805 children aged 0–2 years, because child's length was measured in either lying or standing mode in this age group in the KiGGS data (depending on the child's skills or behaviour), leading to a potential bias in BMI measurements. Further exclusions pertained to 355 children not living with their biological mother, 87 children with missing values on BMI z-score and 1,171 children for whom no information about at least one of the risk factors considered (maternal BMI, maternal smoking in pregnancy, parental SES, exclusive formula-feeding, high TV viewing time) was available, leaving a final dataset of n = 13,223 observations, of which n = 5,986 were 11–17 year-old (this group is also referred to as “teenage children” in the following text).

Quantile regression is a statistical approach of modelling different sample percentiles (‘quantiles’) of an outcome variable with respect to covariates [6], [12], [13], [14]. The approach and interpretation of quantile regression are similar to those of linear regression. While linear regression models the mean of the outcome distribution, quantile regression models selected quantiles, e.g. the 90^th^ percentile (0.90 quantile) - and, like linear regression, uses all available data, irrespective of the percentile modelled. In both cases, regression coefficients quantify potential effects on the specific parameter (mean or quantile) of the outcome distribution on a population level. This means that linear regression coefficients for a binary risk factor can be interpreted as difference of the mean value of the outcome distribution between subjects exposed and not exposed. Similarly, quantile regression coefficients for a binary risk factor represent the difference of the respective quantile in the estimated outcome distribution in subjects exposed vs. not exposed (irrespectively of how many exposed and not exposed subjects lie above or below the respective quantile). Therefore, quantile regression leads to more comprehensive results compared to linear regression because of its ability to assess any part of the outcome distribution. In contrast to logistic regression, quantile regression requires no transformation of the outcome to a binary variable and assesses shifts of specific parts of the continuous outcome distribution instead of probabilities for falling into one outcome category or the other.

We calculated quantile regression models with BMI z-score as outcome variable and considering maternal BMI, maternal smoking in pregnancy, low parental SES, exclusive formula-feeding and high TV viewing time as explanatory variables (thus adjusting for each other), assessing the 0.03, 0.10, 0.20,…, 0.90, and 0.97 quantiles of the BMI z-score distribution. According to European recommendations [15], the 0.90 and 0.97 percentiles can be considered as corresponding to overweight and obesity, respectively. With the exception of maternal BMI (treated as continuous variable), all risk factors were used as binary-coded variables.

Significance of quantile regression effect estimates was derived from 95% confidence intervals calculated by bootstrap methods [13], [16]. We calculated Pearson correlations between quantile regression estimates and the corresponding percentiles to assess potential distribution shifts by the risk factors. To adjust for multiple testing with respect to the five predictors, we considered correlations significant if the respective p-values were p<0.05/5 = 0.01, using Bonferroni's correction method [17]. We compared the median regression coefficients with those for the 0.90 and 0.97 quantile by assessing the differences between the respective estimates for a certain covariate and used the variances of these differences to calculate their 95% confidence intervals (CIs). Additionally, we compared the quantile regression results with those from linear regression models.

To explore potential age effects, all analyses were stratified for 3–10 vs. 11–17 years. A rationale for this cut-off was that German children leave primary school at the age of 10 years and attend a secondary school. Furthermore, information about the 3–10 year-old children was based on measurements and parental questionnaires only, while the 11–17 year-olds received a questionnaire of their own for specific questions, as mentioned above. We performed sensitivity analyses with computer / game pad use as an additional explanatory variable.

All calculations were carried out with the statistical software R 2.6.2 (http://cran.r-project.org), using the *quantreg* package computed by Roger Koenker whose book provides a vignette for the use of this package [13]. In order to avoid bias by selection procedures in the generation of the KiGGS sample, all regression analyses were performed with weighted estimates accounting for the two-staged sample design. The clustering of the children within the primary sample points (communities) was not accounted for in the analysis.

## Results

Although the mean BMI z-score was almost equal in 3–10 and 11–17 year old children, children from the teenage group were more likely (p<0.05) to be exposed to high maternal BMI or exclusive formula-feeding and less likely to have been exposed to maternal smoking in pregnancy compared to the younger children in the dataset (table 1).

**Table 1:** Study characteristics of the data analyzed (n = 13,223).

Variable	3–10 year-old children (n = 7,237)	11–17 year-old children (n = 5,986)	p-value*
	*Mean (SD)*	*Mean (SD)*	
Children's BMI z-score	0.33 (1.12)	0.32 (1.15)	0.84
Age [years]	7.1 (2.3)	14.4 (2.0)	<0.01
Maternal BMI [kg/m^2^]	24.3 (4.6)	24.9 (4.8)	<0.01
	*n (%)*	*n (%)*	
Males	3,679 (50.8%)	3,048 (50.9%)	0.93
High TV viewing time	2,493 (34.4%)	2,071 (34.6%)	0.87
Mother smoking in pregnancy	1,234 (17.1%)	935 (15.6%)	0.03
Low parental SES	1,911 (26.4%)	1,545 (25.8%)	0.45
Exclusively formula fed	1,409 (19.5%)	1,429 (23.9%)	<0.01

*based on two-sample t-test or Fisher's exact test as appropriate.

**Figure 1:**
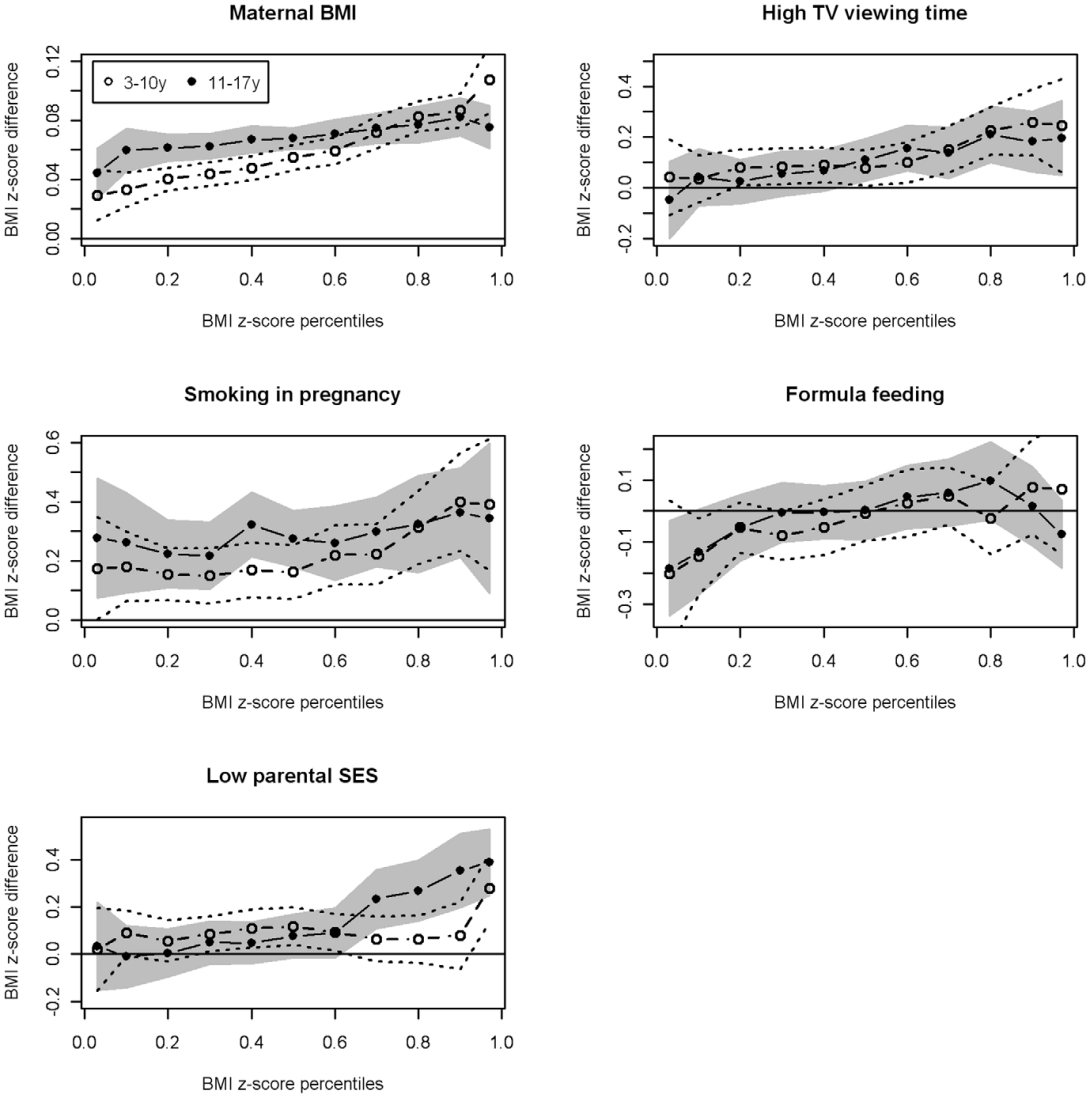
Point estimates and 95% confidence bounds for differences in quantiles of the BMI z-score distribution between children exposed and not exposed to certain risk factors for childhood overweight, stratified by children's age group. In case of maternal BMI, continuous measurements were used. The dots represent specific BMI z-score percentiles (0.03 percentile, 0.1 to 0.9 deciles, and 0.97 percentile) in the multivariable (adjusted) quantile regression model and are connected by dashes to visualize trends by BMI z-score percentiles. The vertical axes vary in order to allow for optimal visualization of the interdependencies of the effects of the respective risk factors and BMI percentile. A horizontal line depicts the y = 0 reference.

The adjusted linear regression estimates for all risk factors except formula-feeding were positive in both the 3–10 year-old and 11–17 year-old children (tables 2 and 3), indicating a shift of the mean BMI in children under exposure. The adjusted quantile regression coefficients were positive for almost any BMI z-score percentile (figure 1), with significant (p<0.05) associations for all percentiles at or above the median, except for exclusive formula-feeding and (partly) low parental SES (tables 2 and 3). This indicates that all risk factors except formula feeding were associated with a shift in BMI z-score to higher values in exposed vs. non-exposed children for medium and high BMI values. However, the regression coefficients for all risk factors examined increased by BMI z-score percentile, and the strongest associations between risk factors and BMI z-score were observed at the upper BMI z-score values (with the exception of the association between formula-feeding and BMI in adolescents).

**Table 2:** Adjusted regression coefficients [±1.96 * standard errors] for risk factors as estimated by linear regression (LR) and quantile regression at specific percentiles (p) in 3–10 year-old children in the KiGGS dataset.

Risk factor	LR	0.03p	0.10p	0.20p	0.30p	0.40p	0.50p	0.60p	0.70p	0.80p	0.90p	0.97p	r*
Maternal BMI [kg/m^2^]	0.06 [±0.01]	0.03 [±0.02]	0.03 [±0.01]	0.04 [±0.01]	0.04 [±0.01]	0.05 [±0.01]	0.05 [±0.01]	0.06 [±0.01]	0.07 [±0.01]	0.08 [±0.01]	0.09 [±0.01]	0.11 [±0.02]	0.97
p-value	<0.001	<0.001	<0.001	<0.001	<0.001	<0.001	<0.001	<0.001	<0.001	<0.001	<0.001	<0.001	<0.001
High TV viewing time	0.13 [±0.05]	0.04 [±0.15]	0.04 [±0.09]	0.08 [±0.07]	0.08 [±0.07]	0.09 [±0.07]	0.08 [±0.07]	0.10 [±0.08]	0.15 [±0.09]	0.22 [±0.09]	0.26 [±0.13]	0.24 [±0.18]	0.93
p-value	<0.001	0.582	0.464	0.023	0.021	0.009	0.031	0.014	0.001	<0.001	<0.001	0.010	<0.001
Smoking in pregnancy	0.22 [±0.06]	0.17 [±0.17]	0.18 [±0.12]	0.15 [±0.09]	0.15 [±0.09]	0.17 [±0.09]	0.16 [±0.09]	0.22 [±0.10]	0.22 [±0.10]	0.31 [±0.12]	0.40 [±0.16]	0.39 [±0.21]	0.84
p-value	<0.001	0.049	0.002	<0.001	0.002	<0.001	<0.001	<0.001	<0.001	<0.001	<0.001	<0.001	<0.001
Exclusive formula feeding	−0.03 [±0.06]	−0.20 [±0.24]	−0.15 [±0.12]	−0.05 [±0.08]	−0.08 [±0.08]	−0.05 [±0.09]	−0.01 [±0.09]	0.03 [±0.11]	0.05 [±0.09]	−0.02 [±0.12]	0.08 [±0.16]	0.07 [±0.21]	0.91
p-value	0.426	0.092	0.019	0.200	0.044	0.260	0.883	0.638	0.299	0.700	0.319	0.514	<0.001
Low parental SES	0.09 [±0.06]	0.02 [±0.18]	0.09 [±0.10]	0.06 [±0.09]	0.09 [±0.07]	0.11 [±0.08]	0.12 [±0.08]	0.09 [±0.08]	0.06 [±0.10]	0.06 [±0.10]	0.08 [±0.14]	0.28 [±0.18]	0.53
p-value	0.005	0.819	0.067	0.208	0.024	0.009	0.004	0.019	0.185	0.220	0.272	<0.001	0.090

*Pearson's correlation coefficients assessing linearity between quantile regression coefficients and the corresponding percentiles of offspring's BMI z-score.

**Table 3:** Adjusted regression coefficients [±1.96 * standard errors] for risk factors as estimated by linear regression (LR) and quantile regression at specific percentiles (p) in 11–17 year-old children in the KiGGS dataset.

Risk factor	LR	0.03p	0.10p	0.20p	0.30p	0.40p	0.50p	0.60p	0.70p	0.80p	0.90p	0.97p	r*
Maternal BMI [kg/m^2^]	0.07 [±0.01]	0.04 [±0.02]	0.06 [±0.01]	0.06 [±0.01]	0.06 [±0.01]	0.07 [±0.01]	0.07 [±0.01]	0.07 [±0.01]	0.07 [±0.01]	0.08 [±0.01]	0.08 [±0.01]	0.08 [±0.01]	0.92
p-value	<0.001	<0.001	<0.001	<0.001	<0.001	<0.001	<0.001	<0.001	<0.001	<0.001	<0.001	<0.001	<0.001
High TV viewing time	0.11 [±0.06]	−0.05 [±0.15]	0.04 [±0.11]	0.02 [±0.09]	0.05 [±0.09]	0.07 [±0.04]	0.11 [±0.08]	0.16 [±0.09]	0.14 [±0.10]	0.21 [±0.11]	0.18 [±0.12]	0.20 [±0.13]	0.96
p-value	<0.001	0.536	0.461	0.580	0.222	0.094	0.009	<0.001	0.007	<0.001	0.003	0.009	<0.001
Smoking in pregnancy	0.30 [±0.08]	0.28 [±0.20]	0.26 [±0.17]	0.22 [±0.11]	0.22 [±0.11]	0.32 [±0.11]	0.27 [±0.09]	0.26 [±0.12]	0.30 [±0.12]	0.32 [±0.08]	0.35 [±0.15]	0.34 [±0.17]	0.74
p-value	<0.001	0.007	0.002	<0.001	<0.001	<0.001	<0.001	<0.001	<0.001	<0.001	<0.001	0.008	0.010
Exclusive formula feeding	−0.01 [±0.07]	−0.18 [±0.15]	−0.13 [±0.14]	−0.05 [±0.11]	0.00 [±0.10]	0.00 [±0.09]	0.00 [±0.09]	0.05 [±0.10]	0.06 [±0.11]	0.10 [±0.12]	0.02 [±0.13]	−0.07 [±0.12]	0.64
p-value	0.693	0.018	0.058	0.316	0.927	0.923	0.953	0.380	0.284	0.126	0.809	0.174	0.033
Low parental SES	0.13 [±0.07]	0.03 [±0.19]	−0.01 [±0.13]	0.00 [±0.10]	0.05 [±0.09]	0.05 [±0.09]	0.08 [±0.09]	0.09 [±0.11]	0.23 [±0.12]	0.27 [±0.13]	0.35 [±0.15]	0.39 [±0.14]	0.93
p-value	<0.001	0.729	0.881	0.928	0.286	0.292	0.103	0.096	<0.001	<0.001	<0.001	<0.001	<0.001

*Pearson's correlation coefficients (r) assessing linearity between quantile regression coefficients and the corresponding percentiles of offspring's BMI z-score.

According to the quantile regression results, the median BMI z-score of teenage children with high TV viewing time, for example, was 0.11 [95% CI: 0.03, 0.19] units higher than the median BMI z-score of children with low TV viewing time (table 3). This risk factor was associated with an average difference of 0.18 [0.06, 0.30] units at the 90^th^ percentile of BMI z-score (difference to median regression coefficient: 0.07 [−0.07, 0.21]) and of 0.20 [0.07, 0.33] units at the 97^th^ percentile (difference to median regression coefficient: 0.09 [−0.08, 0.25]). The linear regression model showed that the mean BMI z-score was 0.11 [0.05, 0.17] units higher when TV viewing time was high (table 3).

Similar results were obtained in the group of the younger children: The median BMI z-score of children with high TV viewing time from this age group was 0.08 [0.01, 0.15] units higher than the median BMI z-score of children with low TV viewing time (table 2). This risk factor was associated with an average difference of 0.26 [0.13, 0.38] units at the 90^th^ percentile of BMI z-score (difference to median regression coefficient: 0.18 [0.03, 0.33]) and of 0.24 [0.06, 0.43] units at the 97^th^ percentile (difference to median regression coefficient: 0.17 [−0.03, 0.37]).

For all variables, the quantile regression coefficients of teenage children increased by percentile rank, with significant correlations (p<0.01) observed for maternal BMI, low parental SES and high TV viewing time (table 3). Similarly, positive associations of quantile regression estimates by percentile rank were found for all variables in 3–10 year-old children, with significant correlations for all risk factors except low parental SES. Inclusion of computer / game pad use as additional explanatory variable did not considerably change the main findings (data not shown).

Figure 1 allows for visual comparison of the quantile regression coefficients for different risk factors by percentile rank in children and adolescents, respectively. While the increase of the quantile regression estimates of low parental SES by percentile rank was more pronounced in teenagers than in younger children, the opposite was true for maternal BMI and smoking in pregnancy. The increase of the quantile regression estimates of formula-feeding and high TV viewing time by percentile rank was weak both for adolescents and younger children.

## Discussion

Our findings demonstrate considerable increases of the regression coefficients of specific risk factors for overweight by BMI z-score percentile rank in children, both in children up to ten years and in teenagers. These results may therefore provide an explanation for the consistently observed trend towards more extreme BMI values in children and adolescents from high-income countries over recent years.

Quantile regression estimates do not depend on the proportion of children exposed in the upper and lower percentiles. Therefore, our findings do not reflect the fact that children who are overweight or obese are more likely to be exposed to certain risk factors, but rather show that these risk factors do not seem to affect the outcome distribution uniformly. As always in a cross-sectional study, we cannot finally preclude common confounding effects by other factors, but we cannot imagine a potential mechanism of residual confounding causing the specific patterns of our main results.

The driving forces of the obesity epidemic in children appear to be high caloric / fat intake and sedentary lifestyle. Media use and TV viewing time as a proxy for sedentary lifestyle have consistently been found to be associated with obesity [18], [19], [20] and seem to have increased over time [21], [22]. Maternal overweight is also on the rise [23], [24]. The higher maternal BMI in the group of older children observed in our study can be explained by a higher age of mothers of 11–17 year-old children compared to mothers of 3–10 year-old children at the time of data collection (41.3 vs. 35.9 years). Maternal age and BMI are known to be slightly positively associated [25], which could be confirmed in our data (r = 0.06, p<0.01).

In general, similar patterns were observed with respect to risk factor regression coefficients by percentile rank in younger and older children. However, subtle differences in the patterns of the two age groups occurred for specific variables: For maternal BMI and smoking in pregnancy, we observed more distinctive patterns with respect to quantile regression estimates at different BMI z-score percentiles in the group of younger children. This appears plausible, since both factors are proxies for the lifestyle of the mother and probably more meaningful at younger age of the offspring. In contrast, low parental SES showed more distinctive patterns across BMI z-score percentiles in adolescents. It is well-known that parental education determines offspring's education to a particular extent in Germany [26], [27]. Secondary school education diversifies around the age of 10 in Germany, so that it appears plausible that the association of low SES and overweight is more pronounced in teenagers than in younger children, with strong effects occurring at the end of adolescence [28]. Since the regression coefficient for low SES was found to increase quite strongly around the 70^th^ percentile, a further explanation could be that higher SES teenagers (or their parents) at these BMI percentiles perceive themselves as overweight and start counteracting their overweight, whereas teenagers with a low SES might also perceive themselves as overweight, but lack the initiative to act against it. It is also possible that high SES teenagers apply the common “beauty ideal” of being slim to themselves, whereas low SES teenagers might perceive themselves as being outside the world of the well-established and “beautiful” in any case, so that the idealistic beauty image represents no role model for them. With respect to high TV viewing time and breastfeeding, no clear age-specific effects on the regression coefficients can be derived from the quantile regression plots.

Our results are likely to be generalisable with respect to other high-income countries. The data were collected within a representative nationwide survey in Germany. In general, temporal trends of childhood overweight are known to be similar in North America and Europe [3], [29].

Since the explanatory variables considered represent established risk factors, the observed associations are likely to be causative, although the cross-sectional design per se does not allow for addressing causal inference [30]. Additionally, all risk factors examined except TV watching, although assessed cross-sectionally by definition, may be interpreted as derived from a retrospective cohort, since the exposures undoubtedly have preceded the outcome. Our results are somewhat similar to those from another study in a different population [14]: That study applied quantile regression on BMI data in adult women and suggested that risk factors for overweight show stronger associations with high compared to low BMI values.

As we have outlined previously, our findings may only partly be explained by dose effects [6]. Instead, genetic variants with a possibly increased susceptibility of carriers to certain risk factors might offer an explanation for differences in the effect magnitude of risk factors by BMI percentiles [4], [6].

Selection bias due to non-responders should not be a major issue with respect to our analyses. The participation rates of 67% were fairly high. BMI calculated from self-reported height and weight did not differ substantially between participants and the 2/3 of non-participants who filled in the short questionnaire [9].

It appears debatable whether BMI adequately reflects overweight status in children and adolescents. For example, overweight children tend to be taller [31] than non-overweight peers. BMI has frequently been used in overweight-related studies, since it can easily be determined from height and weight measurements which are very often routinely taken. Direct measures of body fat mass and fat-free mass might be preferable endpoints to assess the clinical relevance of the impact of risk factors on the obesity epidemic [32].

Unfortunately, our cross-sectional data do not allow identifying target groups for obesity prevention programs, since the examined percentiles refer to the outcome variable BMI z-score at preschool age. Based on these data, it is unclear whether overweight children at school entry would also have been overweight at an earlier age, when a potential intervention (such as breastfeeding) might take place. To quantify differing effects on specific subgroups at the start of an intervention, longitudinal data are required.

In summary, we confirmed that risk factors of childhood overweight are associated with greater shifts in the upper parts of the children's BMI distribution than in the middle and lower parts. These findings pertain also to teenagers and might possibly help to explain the secular shift in the upper BMI percentiles in children and adolescents.

## References

[1] Apfelbacher CJ, Cairns J, Bruckner T, Mohrenschlager M, Behrendt H (2008). Prevalence of overweight and obesity in East and West German children in the decade after reunification: population-based series of cross-sectional studies.. J Epidemiol Community Health.

[2] Armstrong ME, Lambert MI, Sharwood KA, Lambert EV (2006). Obesity and overweight in South African primary school children – the Health of the Nation Study.. S Afr Med J.

[3] Wang Y, Lobstein T (2006). Worldwide trends in childhood overweight and obesity.. Int J Pediatr Obes.

[4] Flegal KM, Troiano RP (2000). Changes in the distribution of body mass index of adults and children in the US population.. Int J Obes Relat Metab Disord.

[5] Kalies H, Lenz J, von Kries R (2002). Prevalence of overweight and obesity and trends in body mass index in German pre-school children, 1982–1997.. Int J Obes.

[6] Beyerlein A, Toschke AM, von Kries R (2010). Risk factors for childhood overweight: shift of the mean body mass index and shift of the upper percentiles: results from a cross-sectional study.. Int J Obes (Lond).

[7] Kautiainen S, Rimpela A, Vikat A, Virtanen SM (2002). Secular trends in overweight and obesity among Finnish adolescents in 1977–1999.. Int J Obes Relat Metab Disord.

[8] Kurth B, Scheidt-Nave C, Schlaud M, Kamtsiuris P, Hölling H (2008). The challenge of comprehensively mapping children's health in a nation-wide health survey: design and first results of the German KiGGS-Study.. BMC Public Health.

[9] Kamtsiuris P, Lange M, Schaffrath Rosario A (2007). [The German Health Interview and Examination Survey for Children and Adolescents (KiGGS): sample design, response and nonresponse analysis].. Bundesgesundheitsblatt Gesundheitsforschung Gesundheitsschutz.

[10] Winkler J, Stolzenberg H (1999). [Social class index in the Federal Health Survey].. Gesundheitswesen.

[11] Cole TJ, Green PJ (1992). Smoothing reference centile curves: the LMS method and penalized likelihood.. Stat Med.

[12] Beyerlein A, Fahrmeir L, Mansmann U, Toschke AM (2008). Alternative regression models to assess increase in childhood BMI.. BMC Med Res Methodol.

[13] Koenker R (2005). Quantile regression.

[14] Terry MB, Wei Y, Esserman D (2007). Maternal, birth, and early-life influences on adult body size in women.. Am J Epidemiol.

[15] Poskitt EM (1995). Defining childhood obesity: the relative body mass index (BMI). European Childhood Obesity group.. Acta Paediatr.

[16] Koenker R, Mandl P, Huskova M (1994). Intervals for regression quantiles.. Asymptotic Statistics.

[17] Bland JM, Altman DG (1995). Multiple significance tests: the Bonferroni method.. BMJ.

[18] Dennison BA, Erb TA, Jenkins PL (2002). Television viewing and television in bedroom associated with overweight risk among low-income preschool children.. Pediatrics.

[19] Andersen RE, Crespo CJ, Bartlett SJ, Cheskin LJ, Pratt M (1998). Relationship of physical activity and television watching with body weight and level of fatness among children: results from the Third National Health and Nutrition Examination Survey.. JAMA.

[20] Robinson TN (1998). Does television cause childhood obesity?. JAMA.

[21] Komlos J, Breitfelder A, Sunder M (2009). The transition to post-industrial BMI values among US children.. Am J Hum Biol.

[22] Christakis DA (2009). The effects of infant media usage: what do we know and what should we learn?. Acta Paediatr.

[23] Heslehurst N, Ells LJ, Simpson H, Batterham A, Wilkinson J (2007). Trends in maternal obesity incidence rates, demographic predictors, and health inequalities in 36,821 women over a 15-year period.. BJOG.

[24] Kim SY, Dietz PM, England L, Morrow B, Callaghan WM (2007). Trends in pre-pregnancy obesity in nine states, 1993–2003.. Obesity (Silver Spring).

[25] Schienkiewitz A, Schulze MB, Hoffmann K, Kroke A, Boeing H (2006). Body mass index history and risk of type 2 diabetes: results from the European Prospective Investigation into Cancer and Nutrition (EPIC)-Potsdam Study.. Am J Clin Nutr.

[26] Gorard S, Smith E (2004). An international comparison of equity in education systems.. Comparative Education.

[27] Jenkins SP, Micklewright J, Schnepf SV (2008). Social segregation in secondary schools: how does England compare with other countries?. Oxford Review of Education.

[28] Toschke AM, Ludde R, Eisele R, von Kries R (2005). The obesity epidemic in young men is not confined to low social classes–a time series of 18-year-old German men at medical examination for military service with different educational attainment.. Int J Obes (Lond).

[29] Kosti RI, Panagiotakos DB (2006). The epidemic of obesity in children and adolescents in the world.. Cent Eur J Public Health.

[30] Toschke AM (2003). Causality and the need of nose length to height curves.. Am J Med Genet A.

[31] Bosy-Westphal A, Plachta-Danielzik S, Dorhofer RP, Muller MJ (2009). Short stature and obesity: positive association in adults but inverse association in children and adolescents.. Br J Nutr.

[32] Toschke AM, Martin RM, von Kries R, Wells J, Smith GD (2007). Infant feeding method and obesity: body mass index and dual-energy X-ray absorptiometry measurements at 9–10 y of age from the Avon Longitudinal Study of Parents and Children (ALSPAC).. Am J Clin Nutr.

